# Insights into the Bioactive Composition, Antioxidant Properties and In Vitro Cell Effects of *Disphyma crassifolium*

**DOI:** 10.3390/foods13081219

**Published:** 2024-04-17

**Authors:** Ana Margarida Silva, Manuela M. Moreira, Filipa Teixeira, Ricardo Ferraz, Miguel Salazar, Cristina Delerue-Matos, Francisca Rodrigues

**Affiliations:** 1 REQUIMTE/LAQV, ISEP, Polytechnic of Porto, Rua Dr. António Bernardino de Almeida 431, 4249-015 Porto, Portugal; ana.silva@graq.isep.ipp.pt (A.M.S.); manuela.moreira@graq.isep.ipp.pt (M.M.M.); 10210131@ess.ipp.pt (F.T.); cmm@isep.ipp.pt (C.D.-M.); 2 Ciências Químicas e Das Biomoléculas (CQB) e Centro de Investigação em Saúde e Ambiente (CISA), Escola Superior de Saúde do Instituto Politécnico do Porto, 4400-330 Porto, Portugal; ricardoferraz@eu.ipp.pt; 3 Agro-On/RiaFresh—Verduras da Ria Formosa, Sítio do Besouro, 8005-421 Faro, Portugal; miguel.salazar@riafresh.pt

**Keywords:** sea fingers, ultrasound-assisted extraction, phenolic profile, pro-healthy effects

## Abstract

*Disphyma crassifolium*, commonly known as sea fingers, is a halophyte plant recently introduced in gourmet cuisine. The present study aims to extract the bioactive compounds of *D. crassifolium* using ultrasound-assisted extraction and employing green solvents (water and ethanol). The antioxidant/antiradical activities, scavenging capacity against reactive species, phenolic profile, and intestinal effects were evaluated. The highest total phenolic (53.13 mg of gallic acid equivalent (GAE)/g on dry weight (dw)) and flavonoid contents (18.98 mg of catechin equivalent (CE)/g dw) as well as antioxidant (149.69 µmol of ferrous sulphate equivalent (FSE)/g dw) and antiradical capacities (9.37 mg of ascorbic acid equivalent (AAE)/g dw) were achieved for the alcoholic extract. Moreover, the alcoholic extract exhibited an efficient uptake of HOCl (IC_50_ = 1.97 µg/mL) and ROO^•^ (0.34 μmol of Trolox equivalent (TE)/mg dw). A total of 34 phenolic compounds were identified in the extracts, with flavonols (isorhamnetin-3-*O*-rutinoside, quercetin-3-*O*-galactoside, and myricetin), flavanols (catechin), and phenolic acids (gallic and ellagic acids) being the principal classes. The intestinal cell viability assays attested that the alcoholic extract presented the lowest IC_50_ values (289.82 and 35.77 µg/mL for HT29-MTX and Caco-2), showing probable anticancer activity. These results emphasize the potential of *D. crassifolium* as a nutraceutical ingredient.

## 1. Introduction

Halophytes constitute a class of plants naturally disseminated in all continents that grow in severe environmental conditions, particularly high levels of salinity. These plants have the capacity to develop as conventional agricultural crops, while in salt stress conditions, they may generate higher amounts of plant biomass. According to Lombardi et al. [1], the optimal conditions for halophyte development range between 50 and 250 mM of NaCl. The plants’ small dimensions, associated with rapid growth, provide a high potential for the hydroponic production of fresh-cut baby leaves that are commonly used in mixed salads, soups, breads, pickles, or as taster ingredients in gourmet food [1]. The adverse environmental conditions where halophytes growth and develop, namely in high levels of salinity, may enhance the production of high levels of bioactive compounds with pro-healthy effects [2]. Nevertheless, few halophytes have been investigated [3,4,5,6,7], and to the best of our knowledge, no studies are available regarding *Disphyma crassifolium*. *D. crassifolium* is a halophyte species found in the supratidal salt marsh that is able to grow at salinities of 1 to 19 ppt under dry conditions [8]. Commonly known as sea fingers, as its structure is similar to human fingers, this halophyte has a prostrate shape, with leaves organized in groups of two or three, forming triangular pyramids [9]. The crispy texture, together with the watery, bitter, and astringent flavor, has attracted the attention of the most recognized cuisine chefs, with it being used in gourmet restaurants [9]. Moreover, sea fingers are commercially reported to be a rich source of protein, fiber, vitamin B6, chromium, β-carotene, and lutein [9]. However, the literature information about the bioactive composition of *D. crassifolium* is scarce. One of the most common techniques employed to study the bioactive composition of plants is ultrasound-assisted extraction (UAE) [10,11,12]. UAE is characterized by a cavitation phenomenon that occurs due to the propagation of ultrasound pressure waves [13], which leads to turbulence, inter-particle collisions, and consequently, perturbation in biomass, accelerating the diffusion process [14]. The bubbles’ implosion generates surface peeling, erosion, and particle breakdown [13]. This phenomenon contributes to the high phenolic recovery observed when compared to conventional extraction techniques. Moreover, the technique’s simplicity, ease of use, and low costs are key factors for its selection. This study intends to evaluate the bioactive composition of *D. crassifolium* with UAE employing the most common and sustainable solvents, namely water and ethanol, in order to understand the pro-healthy properties of sea fingers through the exploration of its phenolic composition, in vitro scavenging properties, and intestinal cell effects.

## 2. Materials and Methods

### 2.1. Chemicals

All reagents were acquired from Sigma-Aldrich (Steinheim, Germany), Merck (Darmstadt, Germany), and Invitrogen Corporation (Life Technologies, S.A., Madrid, Spain). Caco-2 (clone type C2Bbe1) was acquired from American Type Culture Collection (ATCC, Manassas, VA, USA), while HT29-MTX was offered by Dr. T. Lesuffleur (INSERMU178, Villejuif, France).

### 2.2. Samples

*D. crassifolium* leaves were provided by RiaFresh (Faro, Portugal) in September 2022. The samples (*n* = 40) were dried in an Excalibur Food Dehydrator (Sacramento, CA, USA) at 41 °C for 24 h and ground (Moulinex A320, Paris, France) to a particle size of 1 mm, being stored at 4 °C until extraction.

### 2.3. Extraction Procedure 

*D. crassifolium* extracts were obtained via UAE using an ultrasonic probe processor (Sonic Vibracell, model VCX50, Newtown, CT, USA) and a probe tip (No. 630-0219) that was 13 mm in diameter, using the optimal extraction conditions determined by Silva et al. [11], namely a solid–liquid ratio of 10% (*w*/*v*) and an ultrasonic intensity of 30 W/m^2^ for 31.11 min. Water and absolute ethanol were used as solvents. The aqueous extract was frozen at −80 °C for subsequent lyophilization (Telstar, model Cryodos-80, Barcelona, Spain), while the alcoholic one was evaporated at 40 °C (Vaccum Controller V-800, Büchi, Flawil, Switzerland). Samples were kept at room temperature until further analysis.

### 2.4. Total Phenolic and Flavonoid Contents

The total phenolic content (TPC) was measured according to the Folin–Ciocalteu procedure [15], with minor modifications [16], while the total flavonoid content (TFC) was evaluated according to Pinto et al. [17]. Gallic acid (curve linearity range = 5–100 μg/mL; *R*^2^ > 0.998) and catechin (linearity range = 5–300 mg/L; *R*^2^ > 0.990) were, respectively, used as standards for TPC and TFC. The results were expressed as mg of gallic acid equivalent (GAE) per gram of extract on dry weight (dw) (mg GAE/g dw) for TPC and mg of catechin equivalent (CE) per gram of extract on dw (mg CE/g dw) for TFC.

### 2.5. In Vitro Antioxidant/Antiradical Activities

Antioxidant activity was evaluated using the Ferric Reducing Antioxidant Power (FRAP) assay as described by Benzie and Strain [18], with minor modifications [16]. Ferrous sulphate (FeSO_4_·7H_2_O) was used as standard (linearity range: 25–500 μM, *R*^2^ > 0.996). Results were expressed in µmol of ferrous sulphate equivalent (FSE) per gram of extract on dw (µmol FSE/g dw).

Regarding the antiradical activities, the DPPH free radical scavenging potential was performed according to Barros et al. [19], with minor modifications [16], while the ABTS^•+^ radical scavenging activity was based on the procedure described by Re et al. [20]. Trolox (linearity range: 5–175 μg/mL, *R*^2^ > 0.994) and ascorbic acid (linearity range: 5–100 µg/mL; *R*^2^ > 0.991) were, respectively, used as standards for DPPH and ABTS assays. The results were presented as percentage of inhibition (% inhibition) for DPPH assay and mg of ascorbic acid equivalent (AAE) per gram of extract on dw (mg AAE/g dw) for the ABTS procedure.

### 2.6. Reactive Oxygen Species Scavenging Capacity

#### 2.6.1. Superoxide Radical Scavenging Capacity

The superoxide anion radical (O_2_^•−^) scavenging capacity was determined as described by Gomes et al. [21]. The results were expressed as IC_50_ values of the reduction in NBT to a purple-colored diformazan upon reaction with O_2_.

#### 2.6.2. Hypochlorous Acid Scavenging Capacity

The assessment of hypochlorous acid (HOCl) scavenging capacity followed the protocol described by Gomes et al. [21]. The results were presented as the inhibition (IC_50_ values) of HOCl-induced DHR oxidation.

#### 2.6.3. Peroxyl Radical Scavenging Capacity

A peroxyl radical (ROO^•^) assay was conducted in accordance with the procedure outlined by Ou et al. [22]. The outcomes were quantified and presented as micromoles (µmol) of Trolox equivalent (TE) per milligram of extract on a dry weight basis (µmol TE/mg dw).

### 2.7. Phenolic Composition

The phenolic composition of *D. crassifolium* extracts was determined by high-performance liquid chromatography with photodiode array detection (HPLC-PDA), using a Gemini C18 column (250 mm × 4.6 mm, 5 μm) and following the methodology outlined by Moreira et al. [23]. The identification and quantification of the phenolic compounds in aqueous and alcoholic extracts of *D. crassifolium* was carried out via the comparison of the retention time and UV–vis spectra of detected peaks with those obtained for their pure standards. Quantitative results were presented as milligrams (mg) of each phenolic compound per 100 g of extract on a dry weight basis (mg/100 g dw).

### 2.8. Cell Viability Assays

Cell viability assays were carried out utilizing passages 15–16 of Caco-2 and 76–77 of HT29-MTX cells. The 3-(4,5-dimethylthiazol-2-yl)-2,5-diphenyltetrazolium bromide (MTT) assay was performed following the procedure outlined by Pinto et al. [17], employing DMEM and Triton X-100 at 1% (*w*/*v*) as positive and negative controls, respectively. The outcomes were presented as percentages of cell viability.

### 2.9. Statistical Analysis

All measurements were conducted in triplicate, and the outcomes were reported as the mean ± standard deviation (SD) from a minimum of three independent experiments. Statistical analysis was performed using IBM SPSS Statistics 27.0 software (SPSS Inc., Chicago, IL, USA), employing one-way analysis of variance (ANOVA) followed by Tukey’s Honestly Significant Difference (HSD) test with a significance level of *p* < 0.05. Student’s *t*-test was applied to assess significant distinctions between two distinct samples, with a significance level of *p* < 0.05. GraphPad Prism 9 software (GraphPad, La Jolla, CA, USA) was utilized to construct curves illustrating the percentage of inhibition in relation to antioxidant concentration in the context of reactive oxygen species (ROS) scavenging capacity.

## 3. Results and Discussion

### 3.1. TPC, TFC, and In Vitro Antioxidant/Antiradical Activities

The TPC, TFC, and antioxidant/antiradical activities of aqueous and alcoholic extracts of *D. crassifolium* are summarized in Table 1.

As shown in Table 1, the alcoholic extract presented the best results in all spectrophotometric assays. Regarding TPC, the obtained values were 8.49 and 53.13 mg GAE/g dw for the aqueous and the alcoholic extract, respectively, with significant differences (*p* < 0.001) between them. The results achieved for the aqueous extract were similar to the ones reported by Silva et al. [4] for another halophyte, namely *Salicornia ramosissima*, which was extracted via maceration and microwave-assisted extraction (MAE) (15.02 and 8.34 mg GAE/g dw, respectively). In another study, Costa et al. [7] used UAE to extract the halophytes *Crithmum maritimum* and *Salicornia europaea*, employing different ethanol concentrations (0, 10, 20, 40, 80, and 100%; *v*/*v*). The authors achieved TPC values between 3.7 and 23.4 mg GAE/g dw when using 100% and 40% (*v*/*v*) of ethanol as solvents for *C. maritimum*, while for *S. europaea*, the TPC ranged from 1.3 to 9.3 mg GAE/g dw with 100% and 40% or 80% (*v*/*v*) of ethanol, respectively [7]. These values were considerably lower than the ones obtained in the present study. 

Regarding the TFC assay, the alcoholic extract obtained the highest result (18.98 mg CE/g dw), with significant differences (*p* < 0.001) for the aqueous one (8.02 mg CE/g dw). Compared to *S. ramosissima*, *C. maritimum*, and *S. europaea* extracts [4,7], the *D. crassifolium* alcoholic extract presented a higher TFC. 

Regarding the antiradical activities, the alcoholic extract exhibited the highest capacity to scavenge the ABTS^•+^ radical (9.37 mg AAE/g dw), presenting significant differences for the aqueous extract (*p* = 0.012). In contrast, both extracts had similar scavenging capacities for the DPPH free radical, without statistical differences (*p* = 0.216). Compared with the study conducted by Costa et al. [7], the ABTS results were higher than the ones obtained for *S. europaea* extracted with 100% (*v*/*v*) of ethanol. Nevertheless, the results achieved with 0% to 40% (*v*/*v*) of ethanol ranged between 15.1 and 22.8 mg of Trolox equivalent (TE)/g dw [7], respectively, which is higher than the ones reached in the present study. *C. maritimum* extracts also attained better results, namely the extract prepared with 40% (*v*/*v*) of ethanol (59.8 mg TE/g dw) [7]. 

Concerning the antioxidant potential of *D. crassifolium*, the alcoholic extract exhibited the best capacity (149.69 µmol FSE/g dw), with it being significantly superior (*p* < 0.001) to the aqueous extract (11.39 µmol FSE/g dw). Silva et al. reported a lower antioxidant activity for conventional and MAE extracts of *S. ramosissima*, respectively, at 60.61 and 65.56 µmol FSE/g dw [4]. Nonetheless, the results obtained in the present study are in line with the ones reported by Costa et al. [7] for the *S. europaea* extract prepared with 40% of ethanol (*v*/*v*) (143.2 µmol of FeSO_4_∙7 H_2_O/g dw). Therefore, the spectrophotometric analysis performed attested the good antioxidant and antiradical activities of *D. crassifolium* extracts when compared to other halophytes. The differences observed with other studies can be explained by the solvents used, as well as the extraction technique employed. In fact, UAE was demonstrated to be more efficient in the recovery of phenolic compounds when compared to conventional extraction techniques, being characterized by the use of shorter extraction times and temperatures. In addition, the ultrasonic probe is more powerful than the ultrasonic bath, operating at around 20 kHz, which allows for the propagation of ultrasound waves in the extraction media with minimal ultrasonic energy loss. Most importantly, UAE is easily scaled-up and implemented at the industrial level [11].

### 3.2. In Vitro Reactive Oxygen Species Scavenging Capacity

Table 2 summarizes the scavenging potential of *D. crassifolium* extracts against ROS. 

The aqueous extract obtained an IC_50_ = 172.46 µg/mL for the O_2_^•−^ scavenging assay, which is significantly different (*p* < 0.05) from the positive controls employed, namely gallic acid (IC_50_ = 10.39 µg/mL) and catechin (IC_50_ = 26.24 µg/mL). Nevertheless, it was not possible to determine the IC_50_ value for the highest tested concentration (1000 μg/mL) of the alcoholic extract, with an inhibition of 35.95% being determined. The result obtained for the aqueous extract was in line with the ones reported by Correia et al. [5] for the optimal extract of *S. ramosissima* prepared by subcritical water extraction (SWE) (IC_50_ = 158.87 µg/mL). Moreover, the O_2_^•−^ scavenging ability determined for the aqueous extract was higher than the one reported for the *S. ramosissima* extract prepared via conventional extraction (100 °C for 5 min) as well as for the *S. ramosissima* biowaste extracted via maceration (80 °C for 10 min) that were, respectively, IC_50_ = 979.36 μg/mL and IC_50_ = 324.82 μg/mL [4,24].

Concerning the HOCl assay, catechin was the best scavenger (IC_50_ = 0.32 µg/mL), followed by the alcoholic extract (IC_50_ = 1.97 µg/mL), without significant differences (*p* > 0.05) among them. In addition, the aqueous extract presented a good capacity to uptake HOCl, attaining an IC_50_ = 10.48 µg/mL. Compared with the *S. ramosissima* conventional extract, it is perceptible that *D. crassifolium* extracts presented a higher capacity against HOCl. Silva et al. [4] reported an IC_50_ = 90.28 µg/mL for the plant extract, while Pinto et al. [24] obtained an IC_50_ = 27.61 µg/mL for *S. ramosissima* biowaste extract. The IC_50_ value for the *S. ramosissima* optimal extract prepared using SWE was 5.80 µg/mL [5], which is better than the aqueous extract prepared in this study. 

Regarding the uptake of ROO^•^, the increasing order was aqueous extract < alcoholic extract < gallic acid < catechin. The highest result was obtained for catechin (237.11 µmol TE/mg dw), while the lowest was achieved for the aqueous extract (0.08 µmol TE/mg dw). Significant differences (*p* < 0.05) were perceived between catechin and gallic acid as well as between catechin and both *D. crassifolium* extracts. In contrast, no significant differences (*p* > 0.05) were observed between gallic acid and *D. crassifolium* extracts. The present results are slightly higher than the ones reported for *S. ramosissima* conventional and MAE extracts (0.056 and 0.061 µmol TE/mg dw) [4]. Therefore, *D. crassifolium* may constitute an efficient source of antioxidants to neutralize ROS. In addition, the radical scavenging capacity results, namely HOCl and ROO^•^, are generally concordant with the spectrophotometric results (Table 1), highlighting the antiradical potential of the alcoholic extract.

### 3.3. Identification and Quantification of Phenolic Compounds

**Figure 1:**
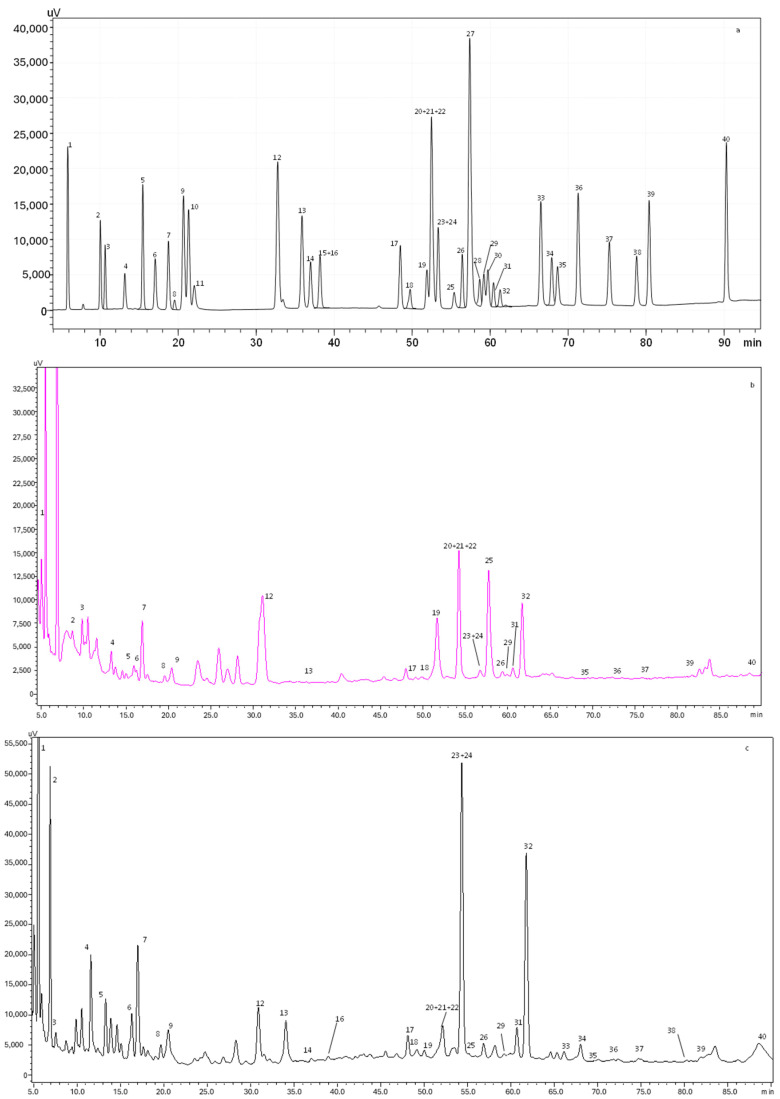
HPLC-PDA chromatogram monitored at 280 nm for (**a**) polyphenol standard mixture of 5 mg/L, and (**b**) aqueous and (**c**) alcoholic extracts of *D. crassifolium*; peak identification: (1) gallic acid, (2) protocatechuic acid, (3) neochlorogenic acid, (4) (+)-catechin, (5) caftaric acid, (6) caffeine, (7) chlorogenic acid, (8) 4-*O*-caffeyolquinic acid, (9) vanillic acid, (10) caffeic acid, (11) syringic acid, (12) (−)-epicatechin, (13) *p*-coumaric acid, (14) trans-ferulic acid, (15) sinapic acid, (16) trans-polydatin, (17) naringin, (18) 3,5-di-caffeoylquinic acid, (19) quercetin-3-*O*-galactoside, (20) resveratrol, (21) quercetin-3-*O*-glucopyranoside, (22) rutin, (23) phloridzin, (24) ellagic acid, (25) 3,4-di-*O*-caffeoylquinic acid, (26) myricetin, (27) cinnamic acid, (28) quercitrin, (29) kaempferol-3-*O*-glucoside, (30) isorhamnetin-3-*O*-glucoside, (31) kaempferol-3-*O*-rutinoside, (32) isorhamnetin-3-*O*-rutinoside, (33) naringenin, (34) trans-epsilon viniferin, (35) quercetin, (36) phloretin, (37) tiliroside, (38) kaempferol, (39) apigenin, and (40) chrysin.

Table 3 summarizes the polyphenols quantified in aqueous and alcoholic extracts of *D. crassifolium*, while Figure 1 shows the chromatograms obtained. 

A total of 34 phenolic compounds were identified and quantified in both extracts. According to Table 3, the alcoholic extract presented the highest amount of phenolic compounds (4150.4 mg/100 g dw), with flavonols being the principal class identified (1612.6 mg/100 g dw), followed by flavanols (984.3 mg/100 g dw). Higher amounts of isorhamnetin-3-*O*-rutinoside (879.5 mg/100g dw), catechin (852.3 mg/100 g dw), and gallic acid (184.4 mg/100g dw) were also observed in the alcoholic extract. Previous studies demonstrated that isorhamnetin is a phenolic compound associated with a high free radical scavenging capacity, promoting the increase in the antioxidant enzyme superoxide dismutase (SOD) and inhibiting the oxidization of low-density lipoprotein [25]. Moreover, catechin is known for its antioxidant and anticancer effects, while gallic acid presents a remarkable radical scavenging potential, regulating the cell signaling pathways and interacting with cancer cell apoptosis [26]. Furthermore, other compounds, such as caffeine and phloridzin, were quantified in the alcoholic extract. It is well established that caffeine intake leads to an increase in antioxidant defenses, with it being able to protect against the oxidative damage of adenine.

In contrast, the aqueous extract presented a total phenolic amount of 639.2 mg/100 g dw. Flavonols (245.9 mg/100 g dw) and phenolic acids (215.2 mg/100 g dw) were the most abundant compounds quantified in the aqueous extract. As stated in Table 3, ellagic acid was the principal phenolic acid quantified (127.0 mg/100 g dw), while myricetin was the main flavonol (106.6 mg/100 g dw). Regarding flavanones, only naringin was identified in the aqueous extract. Recently, Sharifi-Rad et al. [27] described several biological activities of ellagic acid, such as antioxidant, anti-inflammatory, antimutagenic, antiproliferative, antiallergic, cardioprotective, and hepatoprotective properties. Myricetin is another compound that can protect against free radical damages and oxidative stress, neutralizing ROS and inducing the cellular antioxidant enzyme defense system [28].

As expected, the results of TPC and TFC obtained spectrophotometrically for the aqueous (8.49 mg GAE/g dw and 8.02 mg CE/g dw, respectively) and alcoholic (53.13 mg GAE/g dw and 18.98 mg CE/g dw, respectively) extracts were lower than the ones achieved by HPLC-PDA (aqueous extract: 215.2 mg/100 g dw of phenolics and 347.8 mg/100 g dw of flavonoids; alcoholic extract: 929.1 mg/100 g dw of phenolics and 2788.7 mg/100 g dw of flavonoids), despite being consistent since in both analyses the alcoholic extract showed better results. Considering this, it is safe to assume that the use of alcohol as a solvent allowed for the extraction of a greater amount of compound from this natural matrix.

The present results support the richness of *D. crassifolium* alcoholic extract in phenolic compounds when compared to *S. ramosissima* extract prepared by SWE [5]. According to the authors [5], the total amount of compounds quantified by HPLC-PDA varied between 750.13 and 1739.28 mg/100 g dw for samples extracted at 110 °C and 180 °C, respectively. Catechin, chlorogenic acid, and protocatechuic acid were the main compounds quantified in the different extracts, while flavones and flavonols were in smaller amounts [5]. Silva et al. [4] also evaluated the phenolic composition of *S. ramosissima* extracted by maceration and MAE through HPLC-PDA and described the presence of phenolic acids and flavonols, with gallic acid, catechin, epicatechin, rutin, kaempferol-3-*O*-glucoside, and quercetin-3-*O*-galactoside being the principal ones. As previously described, halophyte plants have a high salt stress tolerance that is related to the phenolic composition, with it being responsible for the improvement of antioxidant activity and the reduction in ROS damages, characteristics evidenced in both *D. crassifolium* extracts.

### 3.4. In Vitro Cell Effects

**Figure 2:**
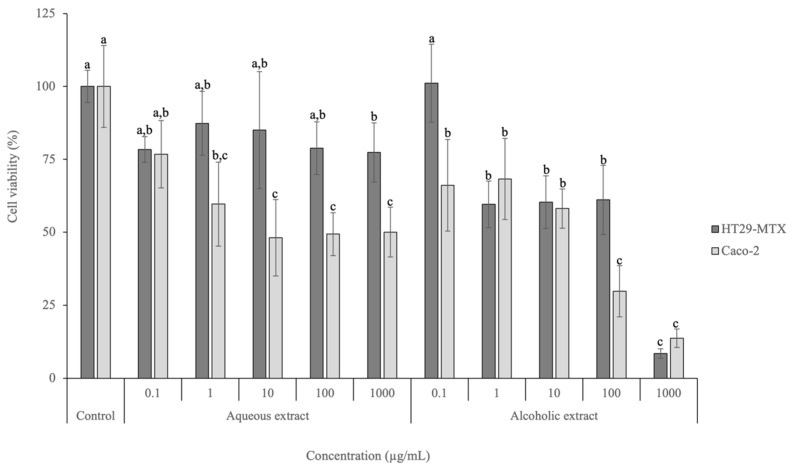
Cell viability effects of aqueous and alcoholic extracts of *D. crassifolium* on HT29-MTX and Caco-2 at the range of concentrations tested (0.1–1000 µg/mL), measured with the MTT assay (*n* = 3). Different letters (a–c) in the same cell line represent significant differences (*p* < 0.05) between different concentrations and the control, according to Tukey’s HSD test.

The viabilities of Caco-2 and HT29-MTX cell lines after exposure to both extracts are represented in Figure 2.

Regarding the HT29-MTX cell line, the lowest concentration tested (0.1 µg/mL) for both extracts did not lead to a viability decrease and did not present significant differences to the positive control employed (*p* > 0.05). Nevertheless, significant differences (*p* < 0.05) were observed between the positive control and the highest concentration tested (1000 µg/mL) for the aqueous extract, achieving a viability of 77.35%. Regarding the alcoholic extract, the viabilities observed for concentrations above 1 µg/mL were significantly different (*p* < 0.05) from the viability of the lowest concentration tested (0.1 µg/mL) and the control. In addition, at the highest tested concentration (1000 µg/mL), the viability was 8.49%. The IC_50_ calculated for HT29-MTX after exposure to the alcoholic extract is 289.82 µg/mL. 

Regarding Caco-2, the highest viability was achieved after exposure to 0.1 µg/mL of the aqueous extract (76.7%). No significant differences (*p* > 0.05) were observed between the concentration of 1 µg/mL and the control. The alcoholic extract led to viabilities between 13.72% (1000 µg/mL) and 68.26% (1 µg/mL), presenting an IC_50_ of 35.77 µg/mL. No significant differences (*p* > 0.05) were observed between 100 and 1000 µg/mL, as well as between 0.1, 1, and 10 µg/mL. 

These results highlight the potential anticancer activity of *D. crassifolium* extracts on intestinal carcinogenic cell lines, probably associated with the phenolic compounds present. Polyphenols are ascribed to several signaling pathways (including p53 and NF-κB) and the restriction of matrix metalloproteinase (MMP) expression (particularly MMP-2 and MMP-9) [29]. Moreover, polyphenols scavenge reactive species that trigger apoptosis by activating signal pathways, including MAPK cascade phosphorylation and PI3K/AKT pathways associated with cell growth and survival, inducing angiogenesis, and stimulating the NF-kB/IL-8 pathway, responsible for cell migration and vessel formation [29]. For example, protocatechuic acid has the capacity to stimulate the c-Jun N-terminal kinase (JNK) and p38-MAPK pathways in different cell lines, such as HepG2 or L-60 leukemia and human gastric adenocarcinoma, leading to cell death, while syringic acid may reduce the expression levels of p53 and BCL-2 proteins and upregulate signaling pathways such as mTOR via AKT [30]. To the best of our knowledge, this is the first study that has assessed the effects of *D. crassifolium* extracts on carcinogenic intestinal cell lines, highlighting their biological potential. Nonetheless, Silva et al. [4] evaluated the cytotoxicity effects of *S. ramosissima* extracts obtained using conventional and MAE methods on HT29-MTX and Caco-2 cell lines. The results revealed that both extracts did not affect the viabilities after exposure to the highest tested concentration (1000 µg/mL) [4]. Similarly, Oueslati et al. [6] studied the effects of *Suaeda fruticosa* extracts, an edible halophyte, obtained using four different solvents (hexane, dichloromethane, methanol, and water) on colon adenocarcinoma cell lines (Caco-2 and HT29). The results showed that the extract prepared with dichloromethane was the most active, exhibiting IC_50_ values of 140 and 12 μg/mL for Caco-2 and HT29 cell lines, respectively [6]. Regarding the aqueous extract, the IC_50_ values were > 200 μg/mL in both cell lines [6]. The authors emphasized that *S. fruticosa* can be a source of anticancer compounds and possibly phenolic compounds, such as protocatechuic acid, syringic acid, and quercetin [6].

## 4. Conclusions

The present study made a step forward on the exploration of the bioactive composition of *D. crassifolium* as a nutraceutical ingredient, using a green and sustainable extraction method. The results demonstrated that *D. crassifolium* extracts, particularly the alcoholic one, exhibited high in vitro antioxidant/antiradical activities, coupled with great amounts of polyphenols (4150.4 mg/100 g dw), particularly isorhamnetin-3-*O*-rutinoside, ellagic acid, gallic acid, catechin, and epicatechin. Moreover, the alcoholic *D. crassifolium* extract was active against colon carcinoma cell lines, despite it being necessary to explore the signaling pathways enrolled in this phenomenon. These results suggest the strong potential of this halophyte as a source of phenolic compounds with pro-healthy properties. As future perspectives, in vitro intestinal permeation assays and in vivo studies should be performed to support the extract’s in vivo efficacy and safety.

## Figures and Tables

**Table 1:** Total phenolic and flavonoid contents (TPC and TFC, respectively) and in vitro antioxidant/antiradical activities evaluated with FRAP, ABTS, and DPPH assays in the aqueous and alcoholic extracts of *D. crassifolium*. The results are presented as mean ± standard deviation (*n* = 3).

*D. crassifolium* Extracts	TPC (mg GAE/g dw)	TFC (mg CE/g dw)	FRAP (µmol FSE/g dw)	DPPH (% Inhibition)	ABTS (mg AAE/g dw)
Aqueous	8.49 ± 0.31	8.02 ± 0.02	11.39 ± 0.56	10.13 ± 1.27	7.73 ± 0.97
Alcoholic	53.13 ± 8.54 *	18.98 ± 1.34 *	149.69 ± 6.79 *	9.12 ± 0.77	9.37 ± 1.11 *

* indicates significant differences between extracts (*p* < 0.05), using Student’s *t*-test analysis.

**Table 2:** Superoxide anion radical (O_2_^•−^), hypochlorous acid (HOCl), and peroxyl radical (ROO^•^) scavenging capacities of the aqueous and alcoholic extracts of *D. crassifolium*. Values are expressed as mean ± standard deviation (*n* = 3). Different letters in the same column indicate significant differences (*p* < 0.05), according to Tukey’s HSD test.

ROS	O_2_^•−^	HOCl	ROO
	IC_50_ (µg/mL)		µmol TE/mg dw
***D. crassifolium* extracts**			
Aqueous	172.46 ± 7.09 ^b^	10.48 ± 0.85 ^c^	0.08 ± 0.01 ^b^
Alcoholic	NA	1.97 ± 0.15 ^a^	0.34 ± 0.04 ^b^
**Positive controls**			
Gallic acid	10.39 ± 1.60 ^a^	5.51 ± 0.34 ^b^	7.51 ± 0.40 ^b^
Catechin	26.24 ± 0.15 ^a^	0.32 ± 0.02 ^a^	237.11 ± 28.37 ^a^

NA: no activity was determined up to the highest tested concentration (1000 μg/mL).

**Table 3:** Identification and quantification of the phenolic compounds present in the aqueous and alcoholic extracts of *D. crassifolium* by HPLC-PDA. Results were expressed as mean ± standard deviations (mg of compound/100 g dw).

Compounds	Aqueous (mg/100g dw)	Alcoholic (mg/100g dw)
Phenolic acids		
Gallic acid	19.2 ± 1.0	184.4 ± 9.2
Protocatechuic acid	21.6 ± 1.1	84.4 ± 4.2
Neochlorogenic acid	4.7 ± 0.2	72.5 ± 3.6
Vanillic acid	3.3 ± 0.2	26.6 ± 1.3
Caffeic acid	ND	ND
Syringic acid	ND	ND
Caftaric acid	11.3 ± 0.6	14.4 ± 0.7
Chlorogenic acid	5.2 ± 0.3	159.7 ± 8.0
4-*O*-caffeyolquinic acid	9.8 ± 0.5	211 ± 11
*p*-Coumaric acid	0.3 ± 0.0	118.7 ± 5.9
Ferulic acid	ND	10.5 ± 0.5
Sinapic acid	ND	ND
3.5-di-caffeoylquinic acid	2.0 ± 0.1	33.4 ± 1.7
Ellagic acid	127.0 ± 6.3	5.2 ± 0.3
4.5-di-*O*-caffeoylquinic acid	10.9 ± 0.5	8.1 ± 0.4
Cinnamic acid	ND	ND
∑Phenolic acids	215.2 ± 10.8	929.1 ± 46.5
Flavanols		
Catechin	33.1 ± 1.7	852.3 ± 42.6
Epicatechin	65.3 ± 3.3	132.0 ± 6.6
∑Flavanols	98.5 ± 4.9	984.3 ± 49.2
Flavanones		
Naringin	1.1 ± 0.1	85.4 ± 4.3
Naringenin	ND	72.3 ± 3.6
∑Flavanones	1.1 ± 0.1	157.7 ± 7.9
Flavonols		
Quercetin-3-*O*-galactoside	86.5 ± 4.3	435.8 ± 21.8
Rutin	0.9 ± 0.0	26.2 ± 1.3
Myricetin	106.6 ± 5.3	137.9 ± 6.9
Kaempferol-3-*O*-glucoside	6.6 ± 0.3	53.1 ± 2.7
Kaempferol-3-*O*-rutinoside	3.6 ± 0.2	9.4 ± 0.5
Quercetin	0.9 ± 0.0	17.4 ± 0.9
Quercitrin	ND	ND
Tiliroside	0.4 ± 0.0	27.1 ± 1.4
Kaempferol	ND	6.6 ± 0.3
Quercetin-3-*O*-glucopyranoside	0.4 ± 0.0	19.7 ± 1.0
Isorhamnetin-3-*O*-glucoside	ND	ND
Isorhamnetin-3-*O*-rutinoside	40.1 ± 2.0	879.5 ± 44.0
∑Flavonols	245.9 ± 12.3	1612.6 ± 80.6
Flavones		
Apigenin	1.0 ± 0.0	8.4 ± 0.4
Chrysin	1.4 ± 0.01	25.7 ± 1.3
∑Flavones	2.3 ± 0.1	34.1 ± 1.7
Others		
Phloridzin	59.7 ± 3.0	140.8 ± 7.0
Phloretin	0.2 ± 0.0	6.6 ± 0.3
Resveratrol	12.9 ± 0.6	1.2 ± 0.1
trans-epsilon viniferin	ND	4.1 ± 0.2
Caffeine	3.32 ± 0.2	273.8 ± 13.7
trans-polydatin	ND	6.1 ± 0.3
∑Others	76.1 ± 3.8	432.6 ± 21.6
∑TOTAL	639.2 ± 32.0	4150.4 ± 207.5

ND: not detected.

## Data Availability

The original contributions presented in the study are included in the article, further inquiries can be directed to the corresponding author.
